# Change to Text of Editorial

**DOI:** 10.1001/jamanetworkopen.2022.55035

**Published:** 2023-01-18

In the Editorial titled “Call for Papers on the Effects of War on Health and Health Care Delivery, Access, and Equity,”^1^ published May 27, 2022, text was added to the fifth paragraph to clarify that authors from the country studied should be included as authors in the manuscript or reasons why none are included should be described.
